# Reshaping Field of View and Resolution with Segmented Reflectors: Bridging the Gap between Rotating and Solid-State LiDARs

**DOI:** 10.3390/s20123388

**Published:** 2020-06-15

**Authors:** Atle Aalerud, Joacim Dybedal, Dipendra Subedi

**Affiliations:** Department of Engineering Sciences, University of Agder, 4879 Grimstad, Norway; joacim.dybedal@uia.no (J.D.); dipendra.subedi@uia.no (D.S)

**Keywords:** LiDAR, environment perception, mirror, point cloud

## Abstract

This paper describes the first simulations and experimental results of a novel segmented Light Detection And Ranging (LiDAR) reflector. Large portions of the rotating LiDAR data are typically discarded due to occlusion or a misplaced field of view (FOV). The proposed reflector solves this problem by reflecting the entire FOV of the rotating LiDAR towards a target. Optical simulation results, using Zemax OpticStudio, suggest that adding a reflector reduces the range of the embedded LiDAR with only 3.9%. Furthermore, pattern simulation results show that a radially reshaped FOV can be configured to maximize point cloud density, maximize coverage, or a combination. Here, the maximum density is defined by the number of mirror segments in the reflector. Finally, a prototype was used for validation. Intensity, Euclidean error, and sample standard deviation were evaluated and, except for reduced-intensity values, no significant reduction in the LiDAR’s performance was found. Conversely, the number of usable measurements increased drastically. The mirrors of the reflector give the LiDAR multiple viewpoints to the target. Ultimately, it is argued that this can enhance the object revisit rate, instantaneous resolution, object classification range, and robustness against occlusion and adverse weather conditions. Consequently, the reflector design enables long-range rotating LiDARs to achieve the robust super-resolution needed for autonomous driving at highway speeds.

## 1. Introduction

LiDAR, an acronym for Light Detection and Ranging, uses laser pulses to take range measurements employing the time-of-flight measurement principle. From these measurements, combined with the LiDAR pose, it is possible to generate a 3D map of the environment. First, the unit sends out a laser pulse, then a sensor on the instrument measures the return signal, and the amount of time it takes for the pulse to bounce back is measured. As light moves at a constant speed, the LiDAR unit can accurately calculate the distance between itself and the target.

Although LiDARs cannot see colors, they have several other properties that favorably apply to automotive and industrial perception systems. Compared to radar, it can obtain much higher spatial resolution owed to the shorter wavelengths, and although it will not see through dense fog, rain, or snow, it performs much better than visible spectrum cameras in adverse weather conditions [1]. Furthermore, while camera triangulation ranging precision falls as the distance to the target increases, LiDAR’s precision can theoretically remain constant [2].

In a presentation [3] at AutoSens, Brussel 2018, Watts states that autonomous cars must be able to separate a person from a bridge column on the highway from a 200 m distance. He explains that this requires, for example, a spatial resolution of 20 cm which corresponds to an angular resolution of 1 mrad or 0.057°. However, no spinning LiDARs today can obtain that high resolution.

Three-dimensional spinning LiDAR scanners that make use of multiple lasers were invented in the mid-2000s. In recent years, however, there has been common opinion that this design—which involves mounting several lasers, for example 8, 16, 32, 64 or 128, onto a rotating gimbal—would soon be rendered obsolete by a new generation of emerging low-cost solid-state LiDAR sensors that use a single stationary laser to scan a scene [4].

As explained in [5], there are three main types of solid-state LiDARs: First, the *flash LiDAR* uses a light source that floods the entire field of view at once, using a pulse like a camera flash. A time-of-flight imager receives that light and calculates a depth image. However, due to cost and eye-safety considerations, range is typically limited. Second, *phased arrays* use a microscopic array of individual antennas. By controlling the timing (phase) between these antennas, the beam can be controlled in a specific direction. The method is promising, but only Quanergy has made sensors using this principle so far. Third, *microelectromechanical system (MEMS) mirrors* are not truly solid-state as there are microscopic moving parts. However, the tiny form factor provides many of the same cost benefits. Here, a single laser (in general) is directed at a spinning mirror to scan a single plane. Furthermore, to add a dimension, a second controlled mirror is needed. Alternatively, a single mirror is actuated around two axes. MEMS mirrors can be susceptible to shock, vibrations and misalignment caused by big changes in temperature. Therefore, the sensor may require recalibration at some point.

The solid-state LiDARs are expected to outperform spinning LiDAR in many applications. However, the classic spinning design still has some advantages. The most obvious one is the omnidirectional horizontal 360° FOV. A LiDAR unit can be placed on the top of a car and get a complete view of a car’s surroundings. Solid-state LiDARs, in contrast, typically have a FOV of 120° or less. It would take at least four solid-state units to achieve coverage comparable to a spinning LiDAR.

Another less obvious advantage is that eye-safety rules allow a moving laser source to emit at a higher power level than a stationary one. With a scanning solid-state unit, putting your eye centimeters from the laser scanner could cause 100% of the laser light to flood into the eye. However, with a spinning sensor, the laser is only focused in any particular direction for a fraction of its 360° rotation. A spinning LiDAR unit can therefore put more power into each laser pulse without creating risk of eye damage. Furthermore, spinning LiDARs typically have much larger apertures compared to solid-state sensors. A larger aperture means more emitted energy and range. The large aperture also makes the sensor less susceptible to “the dead bug problem”, i.e., occlusions from small particles [6]. Hence, spinning units may have a range advantage over stationary ones for at least another decade.

However, if the applications require less than 360° horizontal FOV, using the spinning LiDAR directly will be inefficient as a possibly large portion of the laser beams will be directed away from the target scene and must be discarded. The information carried by the discarded measurements will be wasted and the cost of the useful measurements will be very high. E.g., if only 120° horizontal FOV is needed, applying a spinning LiDAR would discard 67% of the measurements. Using the full capacity of the LiDAR in applications with a need for less than 360° horizontal FOV is therefore a problem worth solving.

In this paper, the solution to the aforementioned problem is to provide a reflector device that maintains the advantages of the spinning LiDAR while providing a reshaped LiDAR beam which may be directed towards a target. Such a LiDAR beam distribution will ensure that all or most of the LiDAR beam is directed towards the target and thus with knowledge also of the beam reflected from the target.

The mirrors of the reflector give the LiDAR multiple viewpoints to the target. Ultimately, it is argued that this can enhance the object revisit rate, instantaneous resolution, object classification range, and robustness against occlusion and adverse weather conditions. Consequently, the reflector design enables long-range rotating LiDARs to achieve the robust super-resolution needed for autonomous driving at highway speeds.

This simple, yet highly effective, reflector device is validated in various configurations using optical simulations, pattern simulations, and experimental prototype results.

## 2. Related Work

It is desirable to use all the data from spinning LiDARs as it is an expensive resource. Even so, a hollow cone comprised of multiple flat mirror segments surrounding the LiDAR to use the full 360° FOV has never been demonstrated in the literature. The concept has only been described by patent applications [7,8] where the latter is part of this work.

However, previous work has used mirrors to redirect LiDAR beams. In [9], Matsubara and Nagatani point out that an omnidirectional LiDAR should be installed on the top of a mobile robot to measure omnidirectionally. However, in this position, the undetectable area close to the robot is often too large. As the authors were instrumenting an inspection robot moving mainly forward, they installed the LiDAR at the front of the robot. However, in this case, the robot body blocked the laser rays behind the sensor, and half of the sensor information that could be acquired originally was invalidated. To use some of the rear-facing LiDAR points, they added two stationary flat mirror segments. Here, the two mirrors are tilted down to increase the vertical FOV of the LiDAR such that objects can be detected in the near vicinity of the vehicle.

Two flat stationary mirrors were also used by [10]. However, here the two mirrors were used in combination with a 2D spinning LiDAR. The assembly was placed on an unmanned aerial vehicle (UAV) to create a constant altitude flight system. The mirrors were placed at the rear end such that the sensor could observe objects in front of the UAV while measuring the vertical distance between the targeted surface and the UAV.

Commonly, rotation is applied to a 2D LiDAR to create a 3D scanner. In [11], the authors constructed a device consisting of a vertical 2D spinning LiDAR mounted between two mirrors in a pan rotational mechanism. The mirrors increased the instant point cloud resolution by reorienting the rear-facing measurements. Furthermore, the pan mechanism enabled the device to obtain a 3D point cloud at high density by scanning the scene to accumulate points. In [12], the authors share their design for both rolling and pitching 3D scanning using a 2D LiDAR. Another tilting scanner using a 3D LiDAR was demonstrated in [13]. Here, the authors proposed a simulation-based methodology to analyze 3D scanning patterns which was applied to investigate the scan measurement distribution produced by a rotating multi-beam LiDAR. A Velodyne VLP-16 was used with the tilting mechanism to create high-resolution 3D scans.

Common for the moving LiDAR scanning applications is the fact that they are not suitable for rapidly changing environments as motion blur will occur. Furthermore, by adding additional moving parts to a sensor, the complexity increases while the robustness decreases. Thus, such scanning solutions may not be applicable in autonomous vehicles moving rapidly in a dynamic environment.

In addition to the possible loss of data from occlusion, it is well documented by [14] that traditional rotating LiDARs have severely reduced performance in adverse weather conditions such as dense fog, rain and turbulent snow. The authors discovered, in a turbulent snow driving experiment, that all tested sensors were blocked by the powder snow and their viewing distance was shortened. One of the tested LiDARs was able to produce points that directly showed the presence of snow, but in the remaining sensors its presence was only observable by missing points in their point clouds.

The Canadian Adverse Driving Conditions (CADC) dataset, presented in [15], contains LiDAR measurements in light, medium, heavy and extreme snowfall conditions. Here, the snowfall condition was classified using Dynamic Radius Outlier Removal (DROR)[16] which is a method for removing snow noise in point cloud data. In [15], Pitropov et. al illustrate that more filtered points correspond to heavier snowfall.

The solid-state LiDAR is commonly considered the next step in LiDAR technology. However, there are currently few qualitative independent publications verifying their actual performance. To the authors’ knowledge, most of the available information origins from specifications that have not been confirmed.

Nevertheless, the advantages of using custom scanning patterns should be considered. For example, the iDAR (intelligent Detection and Ranging) from AEye that does not apply a traditional scanning pattern. Hence, to further promote the benefits of a dynamic scanning pattern, AEye has written a whitepaper proposing a new method of evaluating LiDAR metrics [17]. Here, they emphasize three main metrics: First, they suggest extending the metric of frame rate to include *object revisit rate*, i.e., the time it takes the LiDAR to get successive measurements on an object of interest. The iDAR can revisit an object within the same frame while a spinning LiDAR must typically wait until the next complete scan. Second, they propose to extend the traditional resolution metric to *instantaneous resolution* which describes the degree to which a LiDAR sensor can apply additional resolution to key areas within a frame. Third, they wish to change the focus from object detection range to *object classification range* which is the range where the sensor obtains sufficient data to classify an object.

## 3. Design and Theory

The proposed reflector was designed using a plurality of trapezoid-shaped segments, where the mirrors were arranged on these segments inside a hollow truncated cone. Only flat mirrors were used to avoid divergence of the collimated beam This reflector design allows the centrally placed spinning LiDAR to obtain multiple point of view (POV) measurements of objects placed in zones that are overlapped by multiple mirrors.

**Figure 1:**
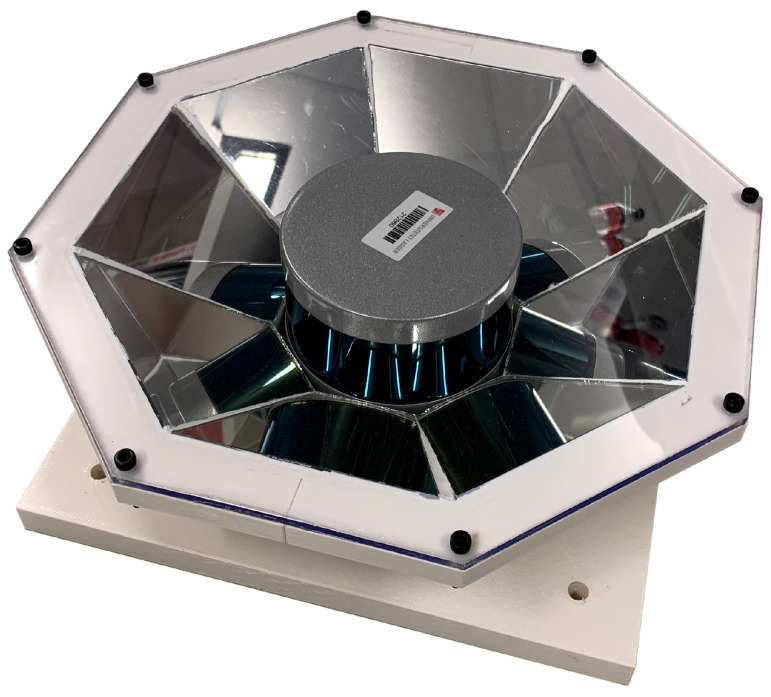
3D printed prototype used in experiments.

The prototype of the reflector, depicted in Figure 1, was printed using the fused deposition modeling (FDM) method and ABS (Acrylonitrile Butadiene Styrene) filament. Reflective segments were cut from a regular 4 mm silver-coated mirror glass and glued inside the printed shape. Here the glue was placed in countersunk cavities to avoid altering mirror angles. Silver-coated mirror glass is a typical second surface mirror, and as explained by [18], in second surface mirrors incident light first passes through a transparent substrate, i.e., the glass, before it is reflected by the coating. This causes chromatic dispersion from the substrate material and ghost reflections on the substrate surface. Consequently, a lower performance was expected compared to a reflector fitted with first surface mirrors. Furthermore, a plexiglass cover was designed to protect the mirrors from dirt and to emulate a glass cover. The complete prototype is 289 mm wide, 88 mm high, and weighs 2470 g. Here, the LiDAR accounts for around 830 g [19].

**Figure 2:**
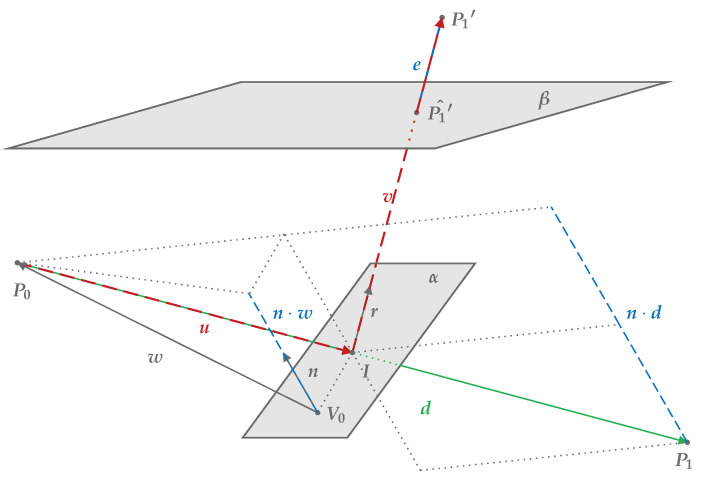
Illustration of vector calculations

To find the transformation from the LiDAR’s observed points to the actual reflected points in the Cartesian space it is first necessary to find the intersection points between laser and mirror. Reflection of a single beam is illustrated in Figure 2 where all the following notations are labeled.

Calculation of intersection between line and plane is based on [20] where the plane is defined by the normal vector, n, and a point on the plane, $V_{0}$. The straight line is given by the points $P_{0}$ and $P_{1}$. The intersection is calculated as

(1) $$\begin{array}{cc} I & =P_{0}+\lambda \left(P_{1}-P_{0}\right) \end{array}$$

where

(2) $$\begin{array}{c} \lambda =-\frac{n\;\cdot \;w}{n\;\cdot \;d}\;\left|\;\begin{array}{c} w=P_{0}-V_{0}\\ d=P_{1}-P_{0} \end{array}\right.\;. \end{array}$$

As shown in Equation (3), the unit reflection vector, r, from a laser coinciding with the mirror plane was calculated by subtracting the vector component that is perpendicular to the mirror plane twice.

(3) $$\begin{array}{cc} r & =\frac{d}{\left|d\right|}-2\frac{d}{\left|d\right|}\;\cdot \;\frac{n}{\left|n\right|} \end{array}$$

Here, d is the full incident beam as described in Equation (2) and n is still the mirror plane normal.

The distance between the transformed point, ${P_{1}}^{\prime }$, and the mirror intersection point, I, is

(4) $$\begin{array}{c} \gamma =|d|-|u| \end{array}\;,$$

where

(5) $$\begin{array}{c} u=I-P_{0} \end{array}\;.$$

Thus, the reflected vector becomes

(6) $$\begin{array}{cc} v & =\gamma r \end{array}$$

and the transformed point is

(7) $$\begin{array}{cc} {P_{1}}^{\prime } & =u+v\;\left|\;P_{0}=0\right. \end{array}$$

**Figure 3:**
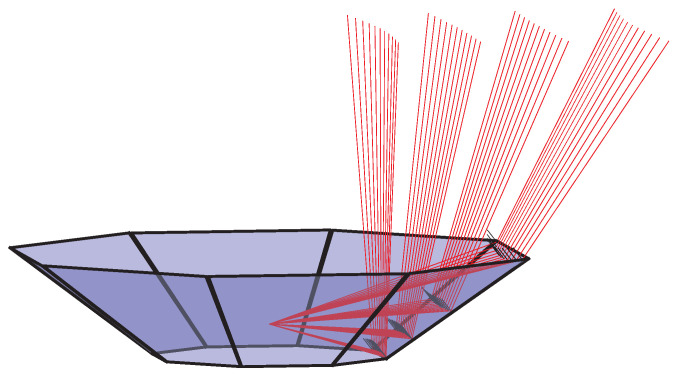
Laser reflections in mirror segment with corresponding surface normals.

To get the transformed position of all measurements, the calculation explained above must be repeated for every measurement of each individual laser in the spinning LiDAR. This process is described by Algorithm 1 where azimuth angle of the LiDAR determines which mirror segment to use for reflection. The function calcTransformedPoint executes the calculations as described by Equatios (1) to (7). To illustrate, a set of reflections are visualized in Figure 3. Here, reflections are shown for four lasers vertically and 11 measurements horizontally on a single reflector segment with corresponding surface normals.
**Algorithm 1** Pseudo code single frame transformation.1:Given sensor position, $P_{0}$2:**for** all m in Mirrors **do**3:    [$V_{0}$, n, **range**] = generateMirrorPlanes(m, tilt, distance)4:**for** all lasers **do**                             ▹ vertical5:    **for** all $P_{1}$ in measurements **do**                    ▹ horizontal6:        **for** all m in Mirrors **do**7:           **if** azimuth ∈ **range then**8:               ${P_{1}}^{\prime }=$ calcTransformedPoint $\left(n,V_{0},P_{0},P_{1}\right)$        ▹ Equatios (1) to (7)

**Figure 4:**
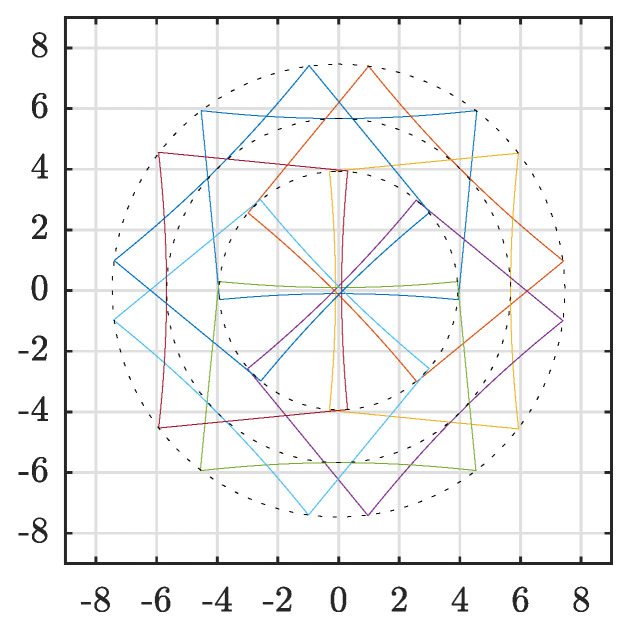
Simulated prototype coverage at 10 m distance. Axes in meter.

The prototype design, as depicted in Figure 1, was built using eight segments and a mirror incline angle of 38°. The LiDAR used with the prototype was the Velodyne VLP-16 [21]. This sensor has 16 lasers placed at 2° intervals to scan a vertical FOV of 30°. Depending on set rotation speed, each laser delivers between 900 and 3600 measurements horizontal points per frame. The LiDAR and reflector configuration is summarized in Table 1. Here, the expected coverage is also shown. FOV columns V, H and MAX describe the resulting coverage limited by the LiDAR’s mirror segments’ vertical, horizontal and maximum field of view, respectively. Here, *N* describes the number of overlapping segments while β is the diametric radial FOV angle. These regions are also shown in Figure 4. Here, the dashed black circles illustrate the different FOV regions while the outline of mirror coverage areas are individually colored.

## 4. Experiments

### 4.1. Power Loss Simulations

To compare the performance of a LiDAR with and without reflector an analysis was performed of the detected scattered light returning from a Lambertian scattering plane using the simulation software Zemax OpticStudio. The analysis was performed using a 10% and 80% reflective Lambertian target, respectively.

The LiDAR chosen as the light source was the OS1-128 from Ouster. As a Zemax Black Box model or detailed laser information was not available, simulation was based on specifications from the (REV:9/5/2019) data sheet [22]. Please note that these specifications are slightly updated in newer revisions. The OS1-128 is assumed to have a linear laser diode array, holding 128 single laser diodes which have different inclination angles. According to the data sheet, the inclination range is −22.5–22.5°, the initial beam radius is 10 mm and the beam divergence is 0.18° FWHM (Full Width at Half Maximum). The analysis was performed using a wavelength of 850 nm with a normalized power of 1 W per beam. It was assumed that scattering analysis would be approximately linearly scaled with the number of beams. Hence, to reduce computation, 5 beams were used for analysis: the maximum inclined, the minimum inclined and three paraxial beams.

An imagined detector was placed by arbitrary choice 20 mm radially away from the central axis of the OS1-128 at the same height as the light source and was configured to have a size of 5×5 mm with $10^{6}$ pixels. This approximation is assumed valid only when comparing reflector losses.

Different reflective materials were evaluated for the reflector. The complete results of this evaluation are shown in Section 5.1, but in general, it will be shown that reflectivity can depend on coating material, the wavelength of the source and the incident angle of the reflection. The Ouster OS1-128 LiDAR uses a laser source in the Near-Infrared (NIR) spectral range at 850 nm while other LiDAR manufacturers commonly use 905 nm or 1550 nm. At these wavelengths standard metal mirror coatings of gold (Au) and silver (Ag) yield high reflectivity. However, the chosen reflection surface used for further analysis was protected silver coating. This is not the material with highest reflectivity, but it represents the most realistic choice given the size of the reflector and subsequent cost and efficiency considerations. The protected silver coating is 0.3 mm thick and consists of three layers, Ag, Al_2_O_3_ and SiO_2_, with the latter two being protective layers against corrosion and abrasion.

**Figure 5:**
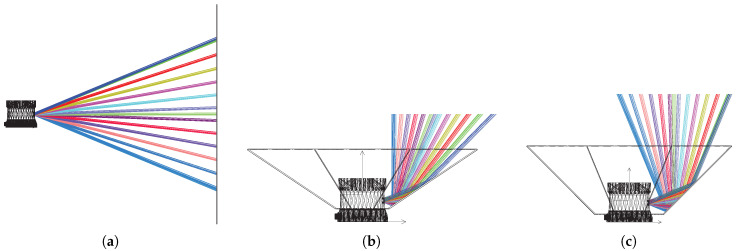
Illustration of the LiDAR and reflector setup for Zemax simulations. Figures show several beams for illustration purpose while five beams were used in the simulations. (**a**) laser beams without reflector. (**b**) laser beams with 33.75° reflector. (**c**) laser beams with 45° reflector.

Zemax simulations were performed for three configurations to evaluate power losses caused by adding a reflector:

- **(A10,A80)** are the baseline simulations without any reflector, as depicted in Figure 5a. Here, the intensity on the detector was measured using a 10% and 80% target for A10 and A80, respectively. The reflective Lambertian target was placed consecutively at 10 m, 50 m and 100 m away from the detector perpendicular to the horizontal plane. Thus, the beams hit the targets with an incident angle between −22.5° and 22.5°.
- **(B10,B80)** are simulations using the same targets and distances as in (A), but here an octagon reflector with an inclination of 33.75° was applied. This setup is depicted in Figure 5b where it is apparent that the bottom laser is reflected perpendicular to the horizontal plane. The targets were placed horizontally above the sensor. Thus, the beams hit the targets with an incident angle between 0° and 45° in this case.
- **(C10,C80)** are also simulations using the same targets and distances as in (A) and a reflector as in (B), but in this case, the reflector inclination was 45°. This setup is depicted in Figure 5c where it is shown that all beams are reflected from horizontal to vertical, but maintaining their relative incident angle regarding the horizontal target plane above the sensor. Therefore, the beams hit the targets with an incident angle between −22.5° and 22.5° similar to (A).

Configurations B and C were expected to have some power loss due to the mirror reflections. In fact, this should be proportional with a reduction of the maximum detection distance. Under the assumptions of an extended target, i.e., a target larger than the laser footprint size, and Lambertian reflectance, a simplified form of a LiDAR range equation can be obtained as described in [23]:

(8) $$\begin{array}{c} P_{r}=\frac{P_{t}D_{r}^{2}\rho }{4R^{2}}\eta_{\mathrm{sys}}\eta_{\mathrm{atm}}\cos\alpha \end{array}$$

Here, $P_{r}$ is the reflected power, $P_{t}$ is the transmitted power, $D_{r}$ is the detector aperture, ρ is the target reflectance, $\eta_{\mathrm{sys}}$ is system and $\eta_{\mathrm{atm}}$ is atmospheric transmission factor and, lastly, α is the angle of incidence between beam and target. Although atmospheric transmission is range dependent, no attenuation is assumed here such that only the effect of the reflector is shown. Hence, assuming that all elements in Equation (8) are constant, except distance from the target, the LiDAR range equation is reduced to

(9) $$\begin{array}{c} P_{r}=\frac{K}{R^{2}} \end{array}$$

where *K* combines the constants such that the inverse range square dependency is revealed. Thus, the range vs. reflected power should follow a $1/R^{2}$ graph, but it must be scaled with a constant value. It follows from this, as further explained in Appendix A, that the expected range loss factor caused by applying the reflector, C, compared to no reflector, A, is

(10) $$\begin{array}{c} R_{\mathrm{loss}}=1-\sqrt{\frac{P_{\mathrm{rC}}}{P_{\mathrm{rA}}}}\;, \end{array}$$

where $P_{\mathrm{rA}}$ and $P_{\mathrm{rC}}$ are the reflected powers, using the same distance, from configuration A and C, respectively.

### 4.2. LiDAR Pattern Simulations

The LiDAR and reflector combination described in Figure 3 has several parameters that affect the LiDAR measurement footprint. Different applications may require different configurations. i.e., one application may require very high resolution in a small region while another seeks to maximize the FOV or a combination. Thus, the experiments described in this section are chosen to highlight key performance differences in this regard. Evaluated parameters of the LiDAR are horizontal and vertical FOV and resolutions. In the same way, number of mirror segments and incline angle relative to the sensor base are evaluated parameters of the reflector. The LiDAR beam divergence was not considered since the simulations were performed using the vector calculations described in Figure 3. Further to the above considerations, distance between mirror segments and the sensor rotation axis was kept constant as this is most likely to be chosen based on practical considerations and not to create different patterns. Currently, only spinning LiDARs are considered to be used in combination with the reflector. Hence, all considered LiDAR specifications have a horizontal FOV of 360°. The pattern simulations are structured in four segments. The first uses different number of mirror segments, the second uses different reflector configurations for a 16 beam LiDAR, similarly, the third segment uses different reflector configurations with a 128-beam LiDAR, and lastly, the fourth segment considers the effect of tilting the target plane.

The first experiment uses a LiDAR with eight laser channels, a vertical FOV of 30° and a horizontal resolution of 900 points. The mirror incline angles were set to 37.5° and the target plane was placed perpendicular to the LiDAR’s rotation axis at a distance of 10 m. Using this configuration, three simulations were executed:

(a) 6 mirror segments (60° horizontal FOV per segment).
(b) 9 mirror segments (45° horizontal FOV per segment).
(c) 12 mirror segments (30° horizontal FOV per segment).

The next three simulations used the same target placement with the 16 beam LiDAR configuration. This had a vertical FOV of 30° and a horizontal resolution of 1800 points. The number of mirror segments was eight, which in turn, yields 45° horizontal FOV per segment. Each simulation used different mirror incline angles:

(d) 37.50° incline angles to maximize FOV without creating a central blind spot.
(e) 41.25° incline angles to create a partial pattern overlap from opposite segments.
(f) 45.00° incline angles to maximize point density by creating complete overlap from opposing segments.

Using the same number of mirror segments and target plane position, the last three simulations were repeated with the 128 beam LiDAR. This had a vertical FOV of 45° and a horizontal resolution on 1024 points. This configuration illustrates a high-resolution case with uniform point density as the vertical and horizontal resolutions are both approximately 0.35°. As the vertical FOV is different, different incline angles were used to illustrate the same principle as for the 16 beam LiDAR:

(g) 33.75° incline angles to maximize FOV without creating a central blind spot.
(h) 39.00° incline angles to create a partial pattern overlap from opposite segments.
(i) 45.00° incline angles to maximize point density by creating complete overlap from opposing segments.

The above pattern experiments all use the same incline angle for all mirror segments. However, this is not a principal limitation as the mirrors can have independent incline angles. Admittedly, this will complicate the reflector design. To illustrate, a simulation was performed using individual mirror angles combined with the LiDAR and target configuration from (d–f).

(j) Individual mirror segment incline angle 45°, 45°, 26.25°, 37.5°, 45°, 37.5°, 26.25° and 45°.
  The last two simulations used the same LiDAR and reflector configuration as described in (d) above. However, to visualize how the target plane angle affect the pattern, the experiment is repeated with two different plane angles:
(k) 30° incident angles between the rotation axis and the target plane normal.
(l) 50° incident angles between the rotation axis and the target plane normal.

All pattern simulation configurations are summarized in Section 5.

### 4.3. Prototype Analysis

**Figure 6:**
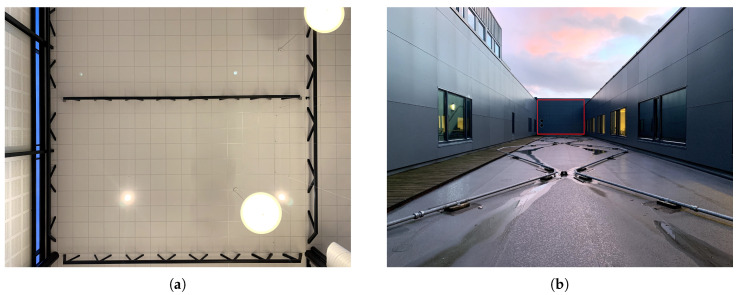
Pictures of test environments. (**a**) Ceiling tiles used for short-range test. (**b**) Wall used for medium range test. Here, the red rectangle indicates the ROI.

The first prototype depicted in Figure 1 was tested with the Velodyne VLP-16 [21] to evaluate the actual consequences of adding a reflector and a transparent plexiglass cover. The following experiments were conducted:

1. *Short-range*: Experiments in an indoor environment, as depicted in Figure 6a, where the target plane was comprised of high reflectance ceiling tiles with a reflectivity of 83% [24]. The (shortest) distance between the LiDAR and the ceiling was 10.3m.
  (a) *Baseline*: A recording of multiple point clouds without using the reflector.
  (b) *Reflector*: A recording of multiple point clouds using the reflector. Here, the LiDAR assembly was placed with the rotational (Z) axis perpendicular to the target plane.
  (c) *Reflector glass*: The same experiment as (1b), but with the addition of the plexiglass.
  (d) *Baseline tilt*: The same recording as (1a) where the point clouds were cropped based on a tilted region of interest (ROI).
  (e) *Tilt reflector*: A recording of multiple point clouds using the reflector. Here, the LiDAR assembly was placed with an incident angle of 30° to the target plane.
  (f) *Tilt reflector glass*: The same experiment as (1e), but with the addition of the plexiglass.
2. *Long-range*: Experiments in an outdoor environment, as depicted in Figure 6b, where the target plane was comprised of gray facade panels with an expected light reflectance value of 14.8% [25]. The distance between the LiDAR and the wall was 35.4m. Recordings were done in clear weather conditions after sunset.
  (a) *Baseline*: A recording of multiple point clouds without using the reflector.
  (b) *Reflector*: A recording of multiple point clouds using the reflector. Here, the LiDAR assembly was placed with the rotational (Z) axis perpendicular to the target plane.
  (c) *Reflector glass*: The same experiment as Figure 2b, but with the addition of the plexiglass.
3. *IR Light Trail*: An experiment to record the actual LiDAR footprint. Here, the reflector was set at a distance of one meter from a white wall in a dark room. A camera, Sony FDR-AXP33, was placed on a tripod behind the top of the reflector and set to record video in infrared mode (night shot) at 25 Hz. While recording, the LiDAR revolution speed was gradually increased to mitigate aliasing. The resulting 2500 images from the video were filtered and fused to create a light trail image of the emitted LiDAR pattern.

For each recording in experiment 1 and 2 a set of frames was selected based on discretized azimuth angles. This was necessary as the Velodyne LiDARs trigger lasers at fixed time intervals and not by using the rotational encoder. Consequently, this may introduce variations of the actual measured point within the set of frames, but as the targets were planes with rather uniform surface, it was found not to influence the metrics significantly. The following metrics were evaluated for each set based on 50 point cloud frames:

- Sum of points detected in at least one frame, n, in the ROI.
- Mean intensity value of the individual point and mean intensity of the ROI, $\bar{i}$.
- Mean Euclidean error of the individual point and mean Euclidean error of the ROI, $\bar{e}$. Here the error, e, was calculated between the transformed point, ${P_{1}}^{\prime }$, and the ground truth estimate, $\hat{{P_{1}}^{\prime }}$, placed on the best fit plane, β, cf. Figure 2.
- Sample standard deviation of measured distance for the individual point, s, and the mean of all s in the ROI, $\bar{s}$.

## 5. Results

### 5.1. Power Loss Simulations

Results of the mirror material analysis are shown in Table 2 for three different wavelengths and four different material types. Protected gold is a standard coating, but was not found in the Zemax coating catalog and was therefore not included in the analysis. Gold has a slightly better reflectivity than silver for all evaluated wavelengths. Protected silver, which was used for simulations, has a reflectivity of 97.15%. This value is close to constant for the evaluated inclination angles 0° to 45°. Two consecutive reflections in protected silver yield an expected loss of 2.85^2^% = 5.8%.

The total power of the scattered light reaching the detector is given in Table 3 for the two different scatter planes, three configurations and three different distances. The total power reaching the detector from the 10% Lambertian scattering plane is about one order of magnitude lower than from the 80% Lambertian scattering plane. Furthermore, Table 3 shows the calculated mean, $\bar{K}$, of the constant part in the LiDAR range equation, cf. Equaton(8). By substituting $\bar{K}$ in Equation (9), the reflected power was calculated as a function of range. RMSE values between the measurements and the $K/R^{2}$ function are shown in the last row of the table.

**Figure 7:**
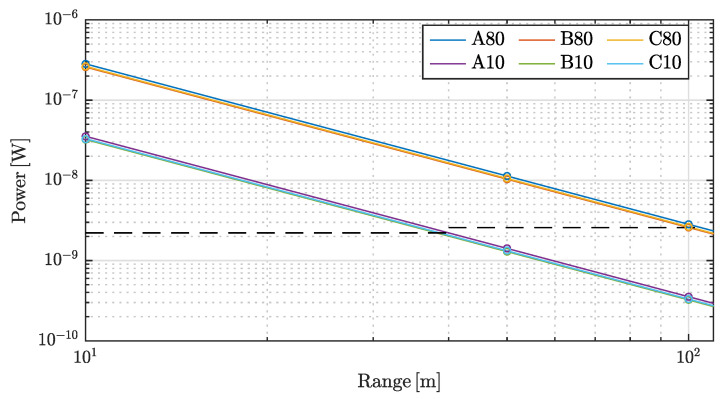
Total scattered power on the detector from Table 3 on logarithmic axes with corresponding $K/R^{2}$ functions. Dashed black lines are calculated lower limits to ensure >90% detection probability for 10% and 80%, respectively.

In Figure 7, total scattered power on the detector is visualized. Both the measured values and the $K/R^{2}$ functions are shown for comparison. Ouster states that their sensor has a range of 105 m on a 80% reflective Lambertian target and a range of 40 m on a 10% reflective Lambertian target, both with >90% detection probability, cf. [22]. The corresponding power levels of this specification, using Equation (9), are shown as horizontal dashed lines.

The losses of detected power caused by the added reflector in configuration B and C compared to the baseline configuration, A, are calculated based on Table 3 and summarized in Table 4. Here it is shown that reflector configuration B yields a loss of 8.2–9.0% and configuration C yields a loss of 6.0–7.6%. Both targets, 10% and 80%, gave similar results.

The expected reduction in range was calculated using Equation (9) and Table 3. The results are summarized in Table 5 where the range reduction for configuration **B** is from 4.2–4.6% while configuration **C** has an expected range reduction of 3.0–3.9%. For the Ouster OS1-128, a loss of 4.6% yields a reduction from 40 m and 105 m to 38.2 m and 100.2 m. Furthermore, a loss of 3.9% yields a reduction to 38.4 m and 100.9 m for the 10% and 80% reflectance case, respectively.

### 5.2. LiDAR Pattern Simulations

**Figure 8:**
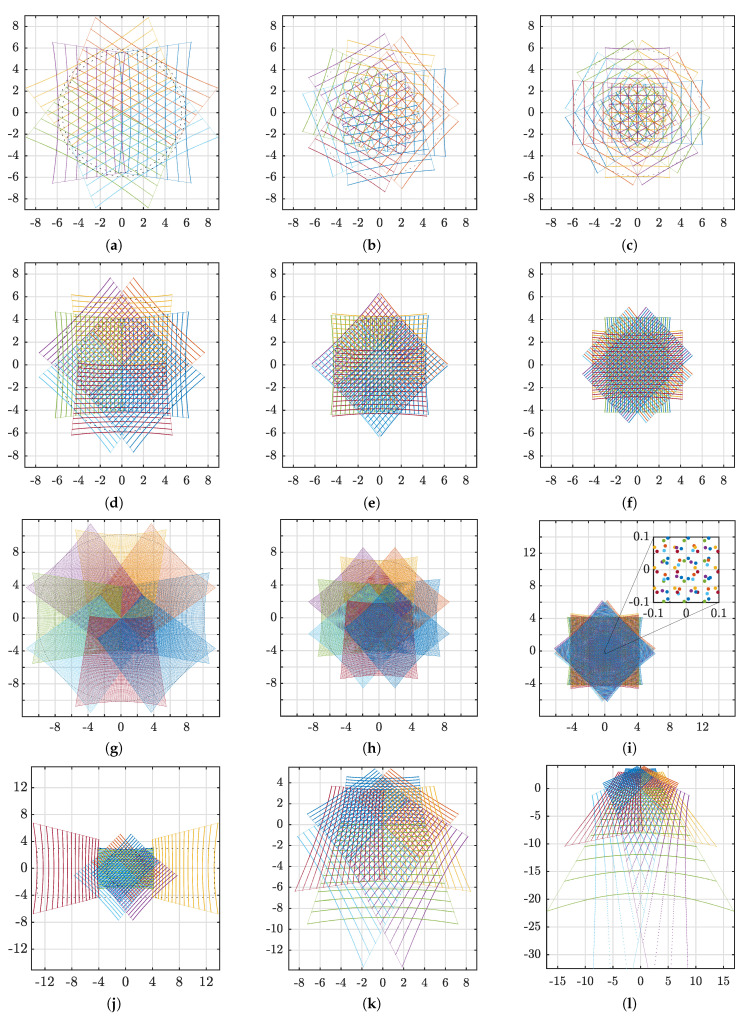
Pattern simulations using different reflector configurations. Colors of LiDAR points are cycled according to reflector segments. A solid colored line outlines each group and dashed black lines show the horizontal, vertical or combined overlapping regions. All figures show the XY-plane in meter. Individual configuration details are listed in Table 6.

Results of the LiDAR pattern simulations are shown in Figure 8 and summarized in Table 6. To clarify, the first column of the table lists the corresponding subfigures. The LiDAR, reflector and target sections give simulator parameters while FOV_V_, FOV_H_ and FOV_HD_ describe the resulting patterns. As the simulations were performed using vectors, no dead zones in mirror intersections were considered. Thus, the reader should be aware that the following results represent an ideal case where the laser footprint consists of a single point which can only hit one mirror at a time.

The LiDAR columns describe vertical (ω) and horizontal (α) field of view, number of laser channels vertically (*p*_V_) and number of measurements horizontally (*p*_H_) per revolution. Furthermore, the reflector column consists of the number of mirror planes, *m*, and mirror incline angle, η. The target configuration is described by the distance (*d*) and the incident angle (γ). Here, the distance was measured from the sensor origin and the target plane along the sensor’s rotation axis. The incident angle was measured between the rotation axis and the target plane normal.

The right part of the table lists the resulting regions and their coverage as shown by dashed circles in Figure 8. FOV_V_ and FOV_H_ describes the regions limited by the LiDAR’s horizontal and vertical FOV, respectively, while FOV_HD_ describes a high definition (HD) region. The diametric radial angle for these regions are shown by β while *N* describes how many reflector segments that overlap the region.

The first three rows of Table 6 correspond to Figure 8a–c. Here, different number of reflector segments are illustrated from simulations using six, nine and twelve segments, respectively. As the number of segments increase, the width of each segment is reduced and, consequently, the smallest region, $\beta_{2}$, shrinks. However, the number of overlaps increase with increasing number of segments.

The remaining simulation results are all focused on the case using eight segments. Here, Figure 8d–f illustrate different reflector incline angles using the LiDAR specifications of a Velodyne VLP-16 at 10 Hz [21]. The 37.50° incline angle used in Figure 8d ensured that the lowest laser beam exited the reflector parallel to the LiDAR rotational axis. Thus, as the figure illustrates, the opposing patterns do not overlap in the center. Instead, the FOV is maximized without creating a central blind spot. The horizontally limited FOV with four overlaps is 43.6° while the vertically limited FOV with at least two overlaps is 61.1°.

In Figure 8e, the incline angle during simulation was 41.25°. It follows that some of the lasers crossed the rotational axis and overlapped with measurements of the opposite mirror. This region, with the maximum of eight overlaps, has a FOV of 14.2°. The remaining vertical FOV corresponds to the horizontal FOV such that both vertically and horizontally limited FOV are close to 45.0° with an overlap of five times.

The HD region was maximized in Figure 8f where the incline angle was 45.0°. In this case, the FOV_HD_ is limited by FOV_V_ which is 31.4° with an overlap of eight times. The remaining horizontally limited FOV_H_ has at least four overlaps.

Similar results are depicted in Figure 8g–i. However, these figures illustrate different reflector configurations using the high-resolution LiDAR configuration where the points have uniform distribution. The first of these three reflector configurations, Figure 8g, used an incline angle of 33.75°. This gives the pattern the same properties as Figure 8d where there is no central overlap, but the FOV is maximized. In this case, the FOV_V_ is 90.9° with one to four overlaps and the FOV_H_ is 41.5° with four overlaps.

The second configuration, Figure 8h, used an incline angle of 39.0°. Consequently, three different density regions are generated. The largest is FOV_V_ with 70.1° and one to four overlaps. The middle region is FOV_H_ with 44.9° and five to seven overlaps. The smallest region is overlapped by all eight patterns creating a high-resolution area with 20.3° FOV.

Configuration Figure 8i used an incline angle of 45.0°, similar to Figure 8f. Thus, the overlap and pattern resolution are maximized. To illustrate this resolution, a small section of the figure is enlarged. Please note that the point sizes do not represent the actual laser footprint as this is expected to be around 40 mm at 10 m distance for this particular LiDAR. As both horizontal and vertical FOV segment are 45°, the combined pattern FOV will also match. In this case, FOV_V_, FOV_H_ and FOV_HD_ are all 46.4° with all eight segments overlapping. Matching horizontal and vertical segment FOV created a pattern with more uniform point distribution as also shown by Figure 8e.

All patterns described above used the same incline angle for the individual mirror segments in each reflector. However, in Figure 8j an example is shown where this is not the case. Here, the mirror angles are 45°, 45°, 26.25°, 37.5°, 45°, 37.5°, 26.25° and 45° listed clockwise from the top. Counting segments in the same order, from one to eight, shows that segments one, two, five and eight are aimed at the center similar to Figure 8f. Segments three and seven increase the horizontal FOV while segments four and six increment the vertical coverage. The resulting FOV, as shown with a black dashed rectangle in Figure 8j, is 107.7° horizontally and 39.6° vertically where the angular range is −23.3° to 16.3°.

The last two illustrations, Figure 8k,l, show the same reflector and LiDAR configuration as Figure 8d. However, in this case, the target plane was tilted 30 and 50°, respectively. The resulting FOV angles remain the same as these are independent from the target positions, but naturally the laser footprints on the target planes are affected.

### 5.3. Prototype Analysis

**Figure 9:**
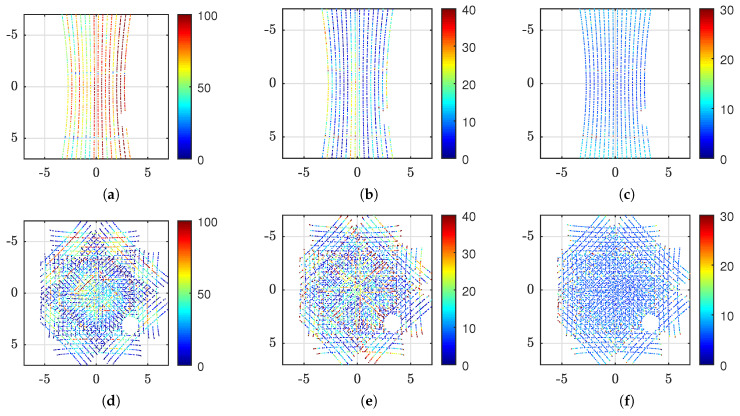
Measurements on 83% reflective ceiling tiles from 10.3 m distance. Axes show the ROI in meter. Here, (**a–c**) show the raw LiDAR measurements in the Z(-Y)-plane while (**d–f**) are measurements of the same ROI using the reflector. These axes correspond to the sensor’s X(-Y)-plane. Furthermore, (**a,d**) show mean intensity using color scale from 0 to 100%, (**b,e**) illustrate mean Euclidean error from best fit plane using color scale in millimeter and (**c,f**) show the sample standard deviation using color scale in millimeter.

**Figure 10:**
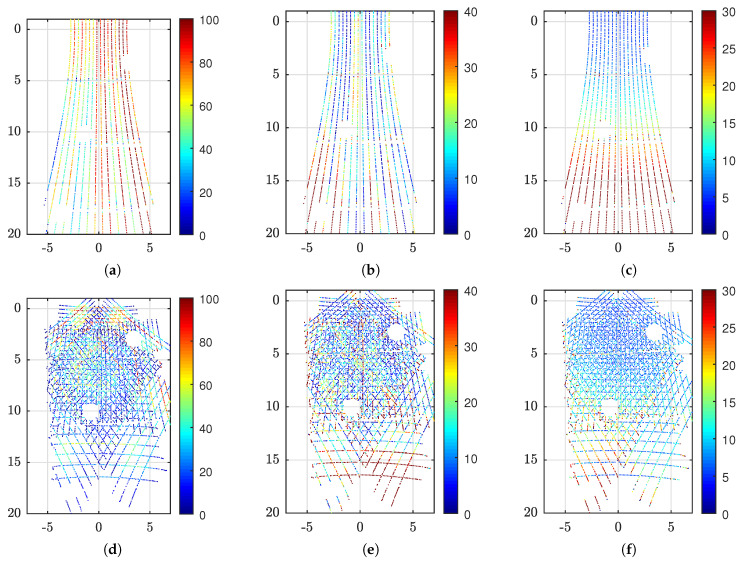
Measurements on 83% reflective ceiling tiles from 10.3 m distance where the reflector is tilted 30° relative to the ceiling. Axes show the ROI in meter on the actual target plane. Here, (**a–c**) show the raw LiDAR measurements in the Z(-Y)-plane of the sensor which is parallel to the ceiling. However, (**d–f**) are measurements using the reflector which has a tilted orientation. For visualization purpose only, these measurements are transformed using a −30° rotation around the sensor X-axis. The resulting points are parallel to the X(-Y)-plane in the LiDAR frame which, in this case, makes it aligned with (**a–c**) for simple comparison. Furthermore, (**a,d**) show mean intensity using color scale from 0 to 100%, (**b,e**) illustrate mean Euclidean error from best fit plane using color scale in millimeter and (**c,f**) show the sample standard deviation using color scale in millimeter.

**Figure 11:**
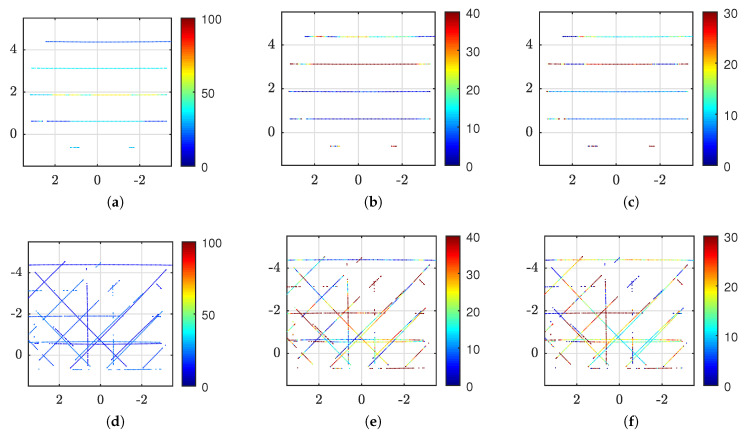
Measurements on exterior facade panels, with expected reflectance of 15%, from 35.4 m distance. Axes show the ROI in meter. Here, (**a–c**) show the raw LiDAR measurements in the (-Y)Z-plane of the sensor while (**d–f**) are measurements in the (-Y)(-X)-plane using the reflector. Furthermore, (**a,d**) show mean intensity using color scale from 0 to 100%, (**b,e**) illustrate mean Euclidean error from best fit plane using color scale in millimeter and (**c,f**) show the sample standard deviation using color scale in millimeter.

The results of the experiments, as described in Section 4.3, are visualized in Figure 9, Figure 10 and Figure 11 and summarized in Table 7.

Moreover, the results from experiment 1a and 1b are shown in Figure 9. The prototype configuration, cf. Table 1, ensures a radial coverage of 42.9° with four overlapping segments, 59.1° with two to four overlapping segments and 73.5° with partial coverage. The ROI selected for comparison was 14.4×14.4 m and included all observable points from the reflector. At a distance of 10.3 m, this corresponds to FOV of 69.9×69.9°. Consequently, this corresponds to a theoretical LiDAR use of 19.4% for the baseline case while 100% of the measurements are inside the ROI for the reflector case. An empty area can be observed around coordinate (3,3) and on the left side in Figure 9d–f. This corresponds to the occluded area behind the suspended lamp and the wall depicted in Figure 6a. As only points near the ceiling plane are included, measurements on the lamp and wall are not shown. The baseline results (Figure 9a–c) contain 5480 points (of 5587 points possible) while the reflector results (Figure 9d–f) contain 23515 points (of 28,800 points possible), i.e., 4.29 times more measurements. Without cropping to the considered plane, the maximum is 5.15 times more measurements for the specified ROI. Figure 9a,d show that the reflector reduces the observed object reflectivity. In this case, the mean intensity value is 73.8% and 31.9% for the baseline and reflector case, respectively. The Euclidean error of the individual measurement is depicted in Figure 9b,e. Here, the baseline result has rather uniform error in the ROI while the reflector has an increased error near the segment intersections. The average errors are 10.0 mm for the baseline and 13.0 mm for the reflector. Furthermore, as shown by Figure 9c,f, the sample standard deviation of the baseline increases with distance while for the reflector it has higher values near the segment intersections. The average standard deviations of all points are 7.0 mm and 6.7 mm for the baseline and reflector case, respectively. Visualization of experiment 1c was found redundant and is omitted from the figure.

Similar to above considerations, Figure 10 visualize the results of experiments 1d and 1e. Here, a new ROI on the target plane is used to match the tilted FOV of the reflector. Due to the tilt, the new ROI covers the larger area of 14.4×21.0 m, but the FOV remains the same as explained above. The occlusion caused by the suspended lamp, cf. Figure 6a, is still present at (3,3) along with two more lamp occlusions and the wall. The remaining measurements after cropping are 5219 points and 21,616 points for the baseline (Figure 10a–c) and reflector (Figure 10d–f), respectively, i.e., 4.14 times more points using the reflector. Intensity, error and standard deviation show similar properties as discussed for Figure 9 above. However, it appears that the sample standard deviation of the reflector, cf. Figure 10f, is less influenced by range than without reflector, cf. Figure 10c. The average standard deviation of Figure 10c is 14.4 mm while for Figure 10f it is 9.0 mm. Visualization of experiment af was found redundant and is omitted from the figure.

The results of long-range experiments 2a,2b are shown in Figure 11. Here, the ROI is only 7×7 m and the LiDAR is placed at a distance of 35.4 m from the target plane. Thus, because the LiDAR was placed far away, and the ROI was small compared to the FOV of the LiDAR, only 441 points appear in the figures of the baseline experiment, see Figure 11a–c. As seen in Figure 11d–f, the reflector increases the number of measurements targeted at the ROI, but there are also several measurements missing. Here, there are 1337 points, which is 3.0 times more than without reflector. It is possible that the low reflectivity of the ROI is near the perceivable limit of the LiDAR at this range. The mean intensity for the baseline case is 33.2% and for the reflector it is 16.0. The Euclidean errors, Figure 11b,e, and sample standard deviations, Figure 11c,f, are somewhat larger with the reflector than the baseline. The mean error is 20.1 mm and 28.5 mm while the mean sample standard deviation is 14.2 mm and 20.1 mm, for baseline and reflector, respectively. Adding the plexiglass at this range, as described by experiment 2c, reduced the amount of measurements to 367 points. This is less than the baseline test as many of the points of the pattern are not detected. Despite more missing measurements, visualization of experiment 1f was found redundant and is omitted from the figure.

Experiments 1 and 2 are summarized in Table 7 with corresponding experiment numbers. Here, the number of points is labeled n. Furthermore, the mean intensity of all points is $\bar{i}$, the mean Euclidean error is $\bar{e}$, and $\bar{s}$ is the mean standard deviation.

**Figure 12:**
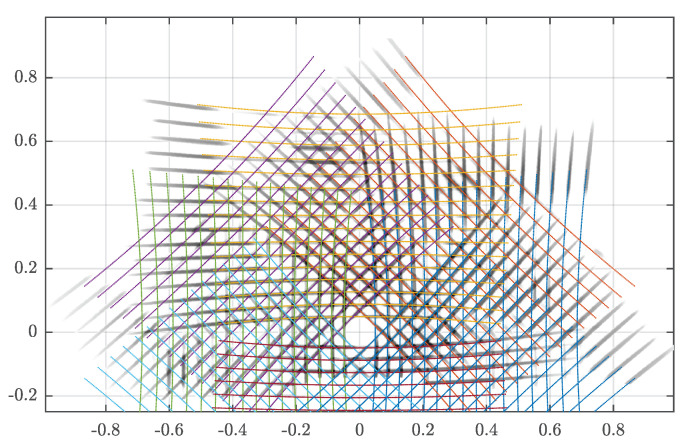
IR light trail pattern depicted in grayscale at a distance of one meter from LiDAR base using the prototype reflector. Darker gray indicates higher light exposure. Colored points show a corresponding simulation. Axes show the YX-plane of the LiDAR in meters.

The resulting light trail of experiment 3 is shown in Figure 12. Here the IR light trail image is shown in grayscale where darker color indicates a higher light exposure. Colored points are simulated LiDAR points using the same parameters as the actual prototype. The recorded light trail has the expected shape and appear to fit well to the simulated pattern, but the calibration accuracy is not currently quantifiable. Nevertheless, it can be observed that areas where LiDAR beams cross have higher intensity.

## 6. Discussion

The loss in power, as presented in Section 5.1, caused by the reflector appears consistent regardless of target reflectivity. Configuration C can describe the losses of the reflector since the beams of this configuration hit the Lambertian scattering plane with the same incident angle as the baseline configuration, A. In configuration C, the total power losses caused by the reflector was 6.0–7.6% where 5.8% was expected from mirror reflections alone. Admittedly, configuration B cannot be compared directly to A due to additional losses caused by a larger inclination angle at the scattering plane. Thus, configuration C must also be used when evaluating range reduction caused by the reflector. Here, the results suggest a loss up to 3.9% which, in turn, translates to a range reduction of 1.6 m and 4.1 m for the 10% and 80% reflectance case, respectively.

Protected silver was used for the analysis as this is a good choice for manufacturing. However, if very high reflectivity is needed, there are dielectric mirror coatings that give at least 99.9% reflection. However, two factors probably inhibit the use of such a dielectric coating. First, these coatings are usually designed for a specific angle of incidence, with degraded performance for all other angles. To account for the wide field of view of the laser, the needed coating, if possible to apply at all, will become very expensive. Second, the shape of the reflector alone is challenging to apply a coating on, even for a pure metal coating. An alternative, for applications that require very high performance, could be to manufacture the 8 panes of the octagon reflector separately, coat them and put them together afterward.

The Zemax analysis was performed based on the Ouster OS1-128 using a wavelength of 850 nm while the prototype used the Velodyne VLP-16 which has a wavelength of 905 nm and a bit lower beam divergence. Furthermore, 905 nm yields a slightly lower reflectivity on protected silver coating. Although a smaller divergence is in principle an advantage, one cannot directly state that the simulated results of Ouster will be true for Velodyne as well. On the other hand, without accurate information about the actual sensor and light source design, simulations must be considered rough estimates. Since the model used for Ouster was just an approximation, one can assume that the interpretation of the results also applies for the Velodyne LiDAR even if the calculated losses may differ a bit.

The pattern simulations shown in Section 5.2 illustrate that the reflector concept can be easily configured to reshape the point cloud density and field of view to accurately fit the desired application’s region of interest.

Increasing the number of segments enables very high density as the maximum number of overlapping patterns is the same as the number of segments. However, the spinning LiDAR’s 360° horizontal FOV is also divided by the number of segments which, in turn, will limit the high-resolution radial FOV of the reflector. For example, if an application requires very high point density, but not a large FOV, it is possible to make a reflector with e.g., 16 segments. Each segment would then have a horizontal FOV of $360^{{}^{\circ}}/16=22.5{}^{{}^{\circ}}$ which would also be the approximate radial FOV of the high-resolution region when using mirrors with 45${}^{{}^{\circ}}$ incline angle. However, a vast number of segments may be undesirable due to the increased number of dead zones between mirror segments, which will, in turn, reduce the number of usable measurements.

On the other hand, if the application requires a high radial FOV, lower mirror incline angles can be used to spread the patterns and, consequently, reducing the overlap. If the mirror incline angles are set lower than $45^{{}^{\circ}}-0.25\;\omega$, cf. Table 6, a blind spot will appear in the middle of the pattern.

As it is possible to make configurations where the mirrors have individual incline angles, one option is to fill the central blind spot by setting one of the mirrors to 45°. This will create a gap in the radial pattern, but allow the rest of the radial FOV to cover a very large area using a single sensor, e.g., by setting seven of the mirrors from Figure 8j to 26.25°.

Reshaping the view is another example where individual mirror angles can be used. As illustrated in Figure 8j, it is possible to tailor the view to the application. This example is well suited for mobile robots or autonomous vehicles. The reflector is here configured to yield high density in the lower central part of the horizon where the relative speed it typically largest. Admittedly, dead zones were not included in the simulations and it is expected that individual incline angles will affect these zones and reduce feasibility of some designs. Further analysis of this is part of future work.

The prototype results presented in Section 5.3 show that the prototype configuration of the reflector concept typically increases point count more than four times after cropping to relevant ROI. Without cropping, the prototype can increase point count more than five times.

Although the point count is higher, Table 7 revealed that the average reported intensity value was reduced with more than 50% when using the reflector. Hence, this vast reduction cannot correspond directly to the power loss. Based on B80 in Table 4, the power loss is expected to be around 8.6%. In fact, Velodyne states in [21]: *The VLP-16 measures reflectivity of an object independent of laser power and distances involved. Reflectivity values returned are based on laser calibration against NIST-calibrated reflectivity reference targets at the factory. Diffuse reflectors report values from 0 to 100 for reflectivities from 0–100%. Retroreflectors report values from 101 to 255, where 255 represents an ideal reflection.* Only the reported values from 0 to 100 were used in the described experiments. As these values are factory calibrated and calculated internally, it is expected that a recalibration is necessary to obtain correct intensity values after adding a reflector.

The average errors and sample standard deviations listed in Table 7 do not indicate that the reflector affect precision of the measurements. However, by investigating Figure 9e,f, it can be found that error increases outside approximately 33.5–40.5° horizontally FOV per segment. Thus, 4.5–11.5° (e.g., $45.0{}^{{}^{\circ}}-40.5{}^{{}^{\circ}}=4.5{}^{{}^{\circ}}$,) should possibly be discarded per intersection depending on the laser channel. The discard width is the same along the mirror intersection, but the relative angle becomes larger closer to the LiDAR. A general formulation to calculate the individual discard angles is part of future work and it is expected that this will depend on the LiDAR beam footprint on the mirror and the individual laser’s distance to the mirror at the intersection.

Given the above, it must be noted that there is a dead zone associated with each mirror intersection. When the laser footprint hits more than one mirror, the measurement must be rejected. However, the dead angle can be reduced by increasing the diameter of the reflector.

The light trail image, in Figure 12, shows that the prototype was built to create the expected transformation. Nevertheless, there is a need for a quantifiable method to evaluate the accuracy of the mirror alignment. Even if the reflections do not reduce the precision of the measurements, a proper mirror calibration method must be part of future work to ensure measurement accuracy. Further to above considerations, an analysis of laser intensity regarding eye-safety regulations should be performed. It can be seen in Figure 12 that the intensity is higher at the LiDAR beam crossing points. Even if the LiDAR inside the reflector is Class 1 eye-safe per IEC 60825-1:2014 [26], it has not yet been validated that the assembly is also eye-safe.

In the introduced literature, both [9,10] used two fixed flat mirrors to reflect LiDAR beams in the same manner as the demonstrated design. Here, [10] used a (single-beam) 2D LiDAR while [9] used a (multi-beam) 3D LiDAR similar to the one demonstrated in this paper. The latter was able to reflect parts of LiDAR beam that would typically be discarded. In comparison, our proposed design can use the full 360° of the LiDAR by deploying multiple fixed mirrors. To the best of the authors’ knowledge, spinning 2D or 3D LiDAR has not been demonstrated with a mirror array of more than two mirrors before.

Although all experiments have shown the abilities of the reflector using 3D LiDAR, this is not a limitation. For example, when the reflector is mounted on a mobile robot in combination with a 2D LiDAR, i.e., spinning LiDAR with one laser, the robot is enabled to perform 3D mapping without the need for the more expensive 3D LiDAR or tilting the sensor. However, the most detailed instantaneous point cloud is obtained using the reflector in combination with a 3D LiDAR. Reflector configurations that create overlapping segment FOV enables multiple segments to measure the same object. When using the LiDAR alone, small obstacles, such as wires or ropes may not be detected, or they may cause an occlusion of the target object. On the contrary, combined with the reflector, smaller objects can be detected due to the higher point density. The occlusions by small obstacles are mitigated as the relative position of the mirrors enables the LiDAR to have multiple POVs to the object.

Another example of small obstacles that create occlusion is snow. In adverse weather conditions, as described by [14], it is assumed likely that a LiDAR combined with the reflector can perform significantly better than the LiDAR alone. No experiments have yet been conducted to validate this, but the expectation arises from the principle of redundant measurements. Typically, if one measurement is blocked by snow, another may pass from a different POV.

On the contrary, in a solid-state LiDAR, all beams exit the sensor in close proximity, i.e., single POV. As the main driver currently is the automotive industry, one of the key value propositions is a small form factor that allows integration into the vehicle design. However, the size of solid-state LiDARs also makes them more susceptible to occlusion on, or in front of, the sensor. This may become a disadvantage for industrial applications where the form is less relevant such as crane and offshore operations.

As stated in [17]: *What matters most is not only how quickly an object can be detected, but how quickly it can be identified and classified, a threat-level decision made, and an appropriate response calculated. A single point detection is indistinguishable from noise. Therefore, we use a common industry definition for detection which involves persistence in adjacent shots per frame and/or across frames. We require five detects on an object per frame (five points at the same range) and/or from frame-to-frame (one single related point in five consecutive frames) to declare that a detection is a valid object.*

Furthermore, the authors stated that: *At 20 Hz, it takes. 25 seconds to define a simple detect.* Although this may be correct considering spinning LiDARs that will not revisit the same object until the next frame, it is no longer true using the reflector. By configuring the reflector with overlap, the object revisit rate can be up to the rotation frequency multiplied with the number of segments. For instance, a LiDAR spinning at 20 Hz using a reflector with eight segments, will have an object revisit rate of 160 Hz in the region with complete overlap. Furthermore, each individual object revisit will have a different POV, which is far more robust than the single POV of the solid-state LiDAR. Thus, a single scan is sufficient for object detection following the definition stated above. Ultimately, one could argue that multiple POVs should also be a system requirement to ensure correct object detection.

The instantaneous resolution, as described in [17] is typically higher for a solid-state LiDAR than a spinning LiDAR within a limited FOV. However, by adding the reflector, the spinning LiDAR can achieve performance beyond a solid-state LiDAR. Similar to the object revisit rate, the instantaneous resolution can reach the multiple of segments when using the reflector. As a result, the object classification range increase accordingly. Given the above considerations, a spinning LiDAR with the proper reflector configuration may prove to be the best choice for a front facing long-range LiDAR for autonomous driving in terms of reliability. Furthermore, the enhanced robustness against adverse weather conditions makes the reflector ideal for industrial outdoor applications such as crane operations, mobile robots, offshore robotics, and personnel safety.

## 7. Conclusions

This paper presents the first simulations and prototype results of a novel LiDAR reflector design that has not been proven in use before.

Optical simulation results, using Zemax OpticStudio, suggest that adding a reflector with protected silver coating will reduce the range of the embedded LiDAR with only 3.9% when target incident angle remains unchanged. Although protected silver coating is a reasonable choice regarding cost and manufacturability, it is possible to use dielectric mirror coatings to increase the range.

The simulated reflector configurations reveal that the reflector can easily be configured to fit different applications. By setting the mirror incline angles to 45°, the resulting pattern will overlap with the number of mirror segments. This will create the highest possible point cloud density. However, by reducing the incline angles, and consequently reducing the pattern overlap, a larger FOV can be obtained. It is also possible to make configurations with individual mirror incline angles, and thus reshape the pattern when a radial footprint is not beneficial.

The considered prototype was constructed for a Velodyne VLP-16 spinning LiDAR with a vertical FOV of 30°. Using reflector mirror incline angles of 38° gave the assembly a radial FOV of 59.1° with full coverage and 73.5° with partial coverage. Comparison with LiDAR alone, using a cropped FOV of 69.9°, gave more than four times more points using the reflector on the plane-cropped ROI. Without cropping, more than five times increase is possible for this ROI.

Prototype experiments also show that the intensity value needs recalibration to yield the correct material reflectivity value. Furthermore, average measurement error and sample standard deviation indicate no precision degradation. However, measurements where the laser footprint hit mirror intersections should be discarded.

The reflected pattern was qualitatively validated using IR light trail photography, but a quantitative calibration and eye-safety analysis remains.

Compared to the current state-of-the-art spinning LiDARs, the reflector can deliver an unprecedented point cloud density. Due to the inherent reflector design, which yields multiple POVs to the same object, it is less susceptible to occlusions from for example snow than for example solid-state LiDARs limited to a single POV.

When combining a reflector with eight segments with a LiDAR spinning at 20 Hz, the object revisit rate can increase to 160 Hz, which traditionally has not been possible using spinning LiDARs. The high object revisit rate makes a single frame sufficient for object detection. The high instantaneous resolution consequently increases the object classification range needed for autonomous driving.

## 8. Patents

The design of the reflector used in this paper is related to “E.P. Patent Application No. PCT/EP2019/085928, Unpublished, (filing date December 18, 2019) (Universitetet i Agder, applicant)” [8].

## Figures and Tables

**Table 1:** LiDAR and reflector configuration used for prototype. The LiDAR vertical and horizontal FOV is given by (ω) and (α), respectively. Furthermore, *p*_V_ and *p*_H_ show the vertical and horizontal resolution. The number of segments in the reflector and mirror incline angle is listed by *m* and η. FOV columns describe the resulting coverage limited by the LiDAR’s segmented vertical, horizontal and maximum field of view, respectively. Here, *N* describes the number of overlapping segments while β is the diametric radial FOV angle.

LiDAR				Reflector		FOV_V_		FOV_H_		FOV_MAX_	
ω [°]	α [°]	*p*_V_	*p*_H_	*m*	η [°]	$\beta_{1}$ [°]	$N_{1}$	$\beta_{2}$ [°]	$N_{2}$	$\beta_{3}$ [°]	$N_{3}$
30	360	16	1800	8	38.00	59.1	2-4	42.9	4	73.5	0-2

**Table 2:** Theoretical reflectivity values of different metal coatings for different relevant LiDAR wavelengths. Assumed thickness of pure metal coatings is 0.2 mm on an acrylic substrate. The protected silver coating is 0.3 mm thick and consists of 3 layers (Ag-Al_2_O_3_-SiO_2_).

λ	Gold [%]	Silver [%]	Copper [%]	Protected Silver [%]
850 nm	97.84	97.28	95.75	97.15
905 nm	98.12	97.49	96.17	96.88
1550 nm	97.16	96.85	96.34	96.53

**Table 3:** Total scattered power on the detector for a 10% and 80% reflective Lambertian target placed 10 m, 50 m and 100 m away from the detector. Reflector design **A** shows values without reflector while design **B** has a tilt of 33.75° and design **C** has a tilt of 45°. Source power is 5 W. $\bar{K}$ is the mean constant part of the LiDAR range equation which is used to calculate reflected power as a function of range, cf. Equation (9). The last row shows the RMSE between the function and measured power.

	A10 [W]	A80 [W]	B10 [W]	B80 [W]	C10 [W]	C80 [W]
10 m	3.54 × 10 ^−8^	2.87 × 10 ^−7^	3.22 × 10 ^−8^	2.59 × 10 ^−7^	3.27 × 10 ^−8^	2.63 × 10 ^−7^
50 m	1.42 × 10 ^−9^	1.13 × 10 ^−8^	1.30 × 10 ^−9^	1.04 × 10 ^−8^	1.34 × 10 ^−9^	1.06 × 10 ^−8^
100 m	3.54 × 10 ^−10^	2.83 × 10 ^−9^	3.25 × 10 ^−10^	2.59 × 10 ^−9^	3.32 × 10 ^−10^	2.64 × 10 ^−9^
$\bar{K}$	3.54 × 10 ^−6^	28.32 × 10 ^−6^	3.24 × 10 ^−6^	25.90 × 10 ^−6^	3.31 × 10 ^−6^	26.38 × 10 ^−6^
RMSE	0.21 × 10 ^−10^	0.67 × 10 ^−10^	0.78 × 10 ^−10^	0.96 × 10 ^−10^	2.24 × 10 ^−10^	3.28 × 10 ^−10^

**Table 4:** Total scattered power loss on the detector, compared to baseline configuration **A**, for a 10% and 80% reflective Lambertian target placed 10 m, 50 m and 100 m away from the detector. Configuration **B** has a tilt of 33.75° and design **C** has a tilt of 45°.

	B10 [%]	B80 [%]	C10 [%]	C80 [%]
10 m	9.0	8.6	7.6	7.0
50 m	8.8	8.6	6.0	6.6
100 m	8.2	8.5	6.3	7.0

**Table 5:** Expected loss in range compared to baseline configuration **A** for a 10% and 80% reflective Lambertian target placed 10 m, 50 m and 100 m away from the detector.

	B10 [%]	B80 [%]	C10 [%]	C80 [%]
10 m	4.6	4.4	3.9	3.6
50 m	4.5	4.4	3.0	3.4
100 m	4.2	4.4	3.2	3.5

**Table 6:** LiDAR and reflector configurations corresponding to Figure 8. The LiDAR vertical and horizontal FOV is given by (ω) and (α), respectively. Furthermore, *p*_V_ and *p*_H_ show the vertical and horizontal resolution. The number of segments in the reflector and mirror incline angle is listed by *m* and η and the target plane distance and angle is shown by *d* and γ. FOV columns describe the resulting coverage limited by the LiDAR’s segmented vertical, horizontal and high definition field of view, respectively. Here, *N* describes the number of overlapping segments while β is the diametric radial FOV angle.

	LiDAR				Reflector		Target		FOV_V_		FOV_H_		FOV_HD_	
	ω [°]	α [°]	p_V_	p_H_	m	η [°]	d [m]	γ [°]	$\beta_{1}$ [°]	$N_{1}$	$\beta_{2}$ [°]	$N_{2}$	$\beta_{3}$ [°]	$N_{3}$
Figure 8a	30	360	8	900	6	37.50	10	0	61.1	2–3	57.9	3	-	-
Figure 8b	30	360	8	900	9	37.50	10	0	61.1	2–3	38.6	4–5	-	-
Figure 8c	30	360	8	900	12	37.50	10	0	61.1	2–5	28.8	6	-	-
Figure 8d	30	360	16	1800	8	37.50	10	0	61.1	2–4	43.6	4	-	-
Figure 8e	30	360	16	1800	8	41.25	10	0	46.5	5	45.0	5	14.2	8
Figure 8f	30	360	16	1800	8	45.00	10	0	31.4	8	45.2	4–6	31.4	8
Figure 8g	45	360	128	1024	8	33.75	10	0	90.9	1–4	41.5	4	-	-
Figure 8h	45	360	128	1024	8	39.00	10	0	70.1	1–4	44.9	5–7	20.3	8
Figure 8i	45	360	128	1024	8	45.00	10	0	46.4	8	46.4	8	46.4	8
Figure 8j	30	360	16	1800	8	multi *	10	0	39.6 *	1–6	107.7 *	1–6	31.4	4–6
Figure 8k	30	360	16	1800	8	37.50	10	30	61.1	2–4	43.6	4	-	-
Figure 8l	30	360	16	1800	8	37.50	10	50	61.1	2–4	43.6	4	-	-

*   Configuration where the mirrors have individual incline angles. These angles are 45, 45, 26.25, 37.5, 45, 37.5, 26.25 and 45° listed clockwise from the top (green) of Figure 8j. FOV angles for multi-configuration indicate vertical and horizontal direction, not radial.

**Table 7:** Prototype results for different configurations as described in Section 4.3. Summarized for the entire ROI; n is the number of points, $\bar{i}$ is the mean intensity, $\bar{e}$ is the mean Euclidean error, and $\bar{s}$ is the mean standard deviation.

Experiment	No.	*n*	$\bar{i}$	$\bar{e}$ [mm]	$\bar{s}$ [mm]
Baseline	1a	5480	73.8	10.0	7.0
Reflector	1b	23515	31.9	13.0	6.7
Reflector Glass	1c	21153	23.4	11.4	6.2
Baseline tilt	1d	5219	70.7	15.0	14.4
Tilt reflector	1e	21616	28.8	15.6	9.0
Tilt reflector glass	1f	19311	23.6	14.0	8.0
Baseline	2a	441	33.2	20.1	14.2
Reflector	2b	1337	16.0	28.5	20.1
Reflector glass	2c	367	11.5	28.7	20.5

**Figure A1:**
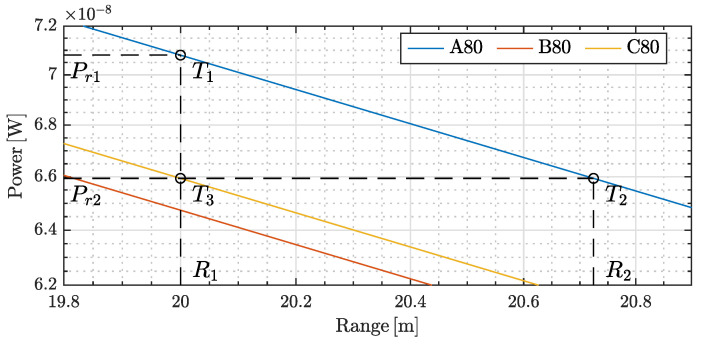
An enhanced and labeled section of Figure 7 showing total scattered power on the detector as a function of range. Both axes are logarithmic.

## Appendix A

This section explains the calculation to obtain Equation (10) using Equation (9). In Figure A1, a section of Figure 7 is shown with additional labeled points. The figure shows the functions $P_{r}=K/R^{2}$ using *K* from three of the configuration results in Table 3.

Application of Equation (9) in point $T_{1}\left(R_{1},P_{r1}\right)$ yields

(A1) $$\begin{array}{ccc} P_{r1} & =\frac{K_{A}}{{R_{1}}^{2}}\;\Rightarrow \;K_{A} & =P_{r1}\;{R_{1}}^{2}\;, \end{array}$$

where $K_{A}$ is a constant. At point $T_{2}\left(R_{2},P_{r2}\right)$ it yields

(A2) $$\begin{array}{ccc} P_{r2} & =\frac{K_{A}}{{R_{2}}^{2}}\;\Rightarrow \;K_{A} & =P_{r2}\;{R_{2}}^{2}\;, \end{array}$$

where $K_{A}$ is a constant. At point $T_{3}\left(R_{1},P_{r2}\right)$ it yields

(A3) $$\begin{array}{ccc} P_{r2} & =\frac{K_{C}}{{R_{1}}^{2}}\;\Rightarrow \;K_{C} & =P_{r2}\;{R_{1}}^{2}\;, \end{array}$$

where $K_{C}$ is a constant. Combining Equations (A2) and (A3) yields

(A4) $$\begin{array}{ccc} \frac{K_{A}}{{R_{2}}^{2}} & =\frac{K_{C}}{{R_{1}}^{2}}\;\Rightarrow \;\frac{{R_{1}}^{2}}{{R_{2}}^{2}} & =\frac{K_{C}}{K_{A}}\;. \end{array}$$

Substituting from Equations (A1) and (A3) in Equation (A4) yields

(A5) $$\begin{array}{ccc} \frac{{R_{1}}^{2}}{{R_{2}}^{2}} & =\frac{P_{r2}\;{R_{1}}^{2}}{P_{r1}\;{R_{1}}^{2}}=\frac{P_{r2}}{P_{r1}}\;\Rightarrow \;\frac{R_{1}}{R_{2}} & =\sqrt{\frac{P_{r2}}{P_{r1}}}\;. \end{array}$$

Thus, it follows that the relation between return power ratio and range ratio between configurations are

(A6) $$\begin{array}{cc} \frac{R_{C}}{R_{A}} & =\sqrt{\frac{P_{\mathrm{rC}}}{P_{\mathrm{rA}}}}\;, \end{array}$$

where $R_{A}$ and $R_{C}$ can be any range with the same returned power along configuration A80 and C80, respectively. Similarly, $P_{\mathrm{rA}}$ and $P_{\mathrm{rC}}$ can be any returned power at the same range along configuration A80 and C80. Conclusively, it follows that the relative range loss between configuration A80 and C80 is

(A7) $$\begin{array}{c} R_{\mathrm{loss}}=1-\frac{R_{C}}{R_{A}}=1-\sqrt{\frac{P_{\mathrm{rC}}}{P_{\mathrm{rA}}}}\;. \end{array}$$
